# Examining the transcriptional response of overexpressing anthranilate synthase in the hairy roots of an important medicinal plant *Catharanthus roseus* by RNA-seq

**DOI:** 10.1186/s12870-016-0794-4

**Published:** 2016-05-06

**Authors:** Jiayi Sun, Harish Manmathan, Cheng Sun, Christie A. M. Peebles

**Affiliations:** Chemical and Biological Engineering Department, Colorado State University, Campus delivery 1370, Fort Collins, 80523 USA; Soil and Crop Sciences Department, Colorado State University, Campus deliver 1170, Fort Collins, Colorado 80523 USA; Department of biology, Colorado State University, 1878 Campus Delivery, Fort Collins, Colorado 80521 USA; Key Laboratory of Pollinating Insect Biology of the Ministry of Agriculture, Institute of Apicultural Research, Chinese Academy of Agriculture Science, Beijing, 10093 China

**Keywords:** Terpenoid indole alkaloid, Madagascar periwinkle, Transcription factors, Plant secondary metabolism, High-throughput sequencing, Plant stress response

## Abstract

**Background:**

Clinically important anti-cancer drugs vinblastine and vincristine are solely synthesized by the terpenoid indole alkaloid (TIA) pathway in *Catharanthus roseus*. Anthranilate synthase (AS) is a rate-limiting enzyme in the TIA pathway. The transgenic *C. roseus* hairy root line overexpressing a feedback insensitive ASα subunit under the control of an inducible promoter and the ASβ subunit constitutively was previously created for the overproduction of TIAs. However, both increases and decreases in TIAs were detected after overexpressing ASα. Although genetic modification is targeted to one gene in the TIA pathway, it could trigger global transcriptional changes that can directly or indirectly affect TIA biosynthesis. In this study, Illumina sequencing and RT-qPCR were used to detect the transcriptional responses to overexpressing AS, which can increase understanding of the complex regulation of the TIA pathway and further inspire rational metabolic engineering for enhanced TIA production in *C. roseus* hairy roots.

**Results:**

Overexpressing AS in *C. roseus* hairy roots altered the transcription of most known TIA pathway genes and regulators after 12, 24, and 48 h induction detected by RT-qPCR. Changes in the transcriptome of *C. roseus* hairy roots was further investigated 18 hours after ASα induction and compared to the control hairy roots using RNA-seq. A unigene set of 30,281 was obtained by *de novo* assembly of the sequencing reads. Comparison of the differentially expressed transcriptional profiles resulted in 2853 differentially expressed transcripts. Functional annotation of these transcripts revealed a complex and systematically transcriptome change in ASαβ hairy roots. Pathway analysis shows alterations in many pathways such as aromatic amino acid biosynthesis, jasmonic acid (JA) biosynthesis and other secondary metabolic pathways after perturbing AS. Moreover, many genes in overall stress response were differentially expressed after overexpressing ASα.

**Conclusion:**

The transcriptomic analysis illustrates overexpressing AS stimulates the overall stress response and affects the metabolic networks in *C. roseus* hairy roots. The up-regulation of endogenous JA biosynthesis pathway indicates the involvement of JA signal transduction to regulate TIA biosynthesis in ASαβ engineered roots and explained why many of the transcripts for TIA genes and regulators are seen to increase with AS overexpression.

**Electronic supplementary material:**

The online version of this article (doi:10.1186/s12870-016-0794-4) contains supplementary material, which is available to authorized users.

## Background

**Fig. 1:**
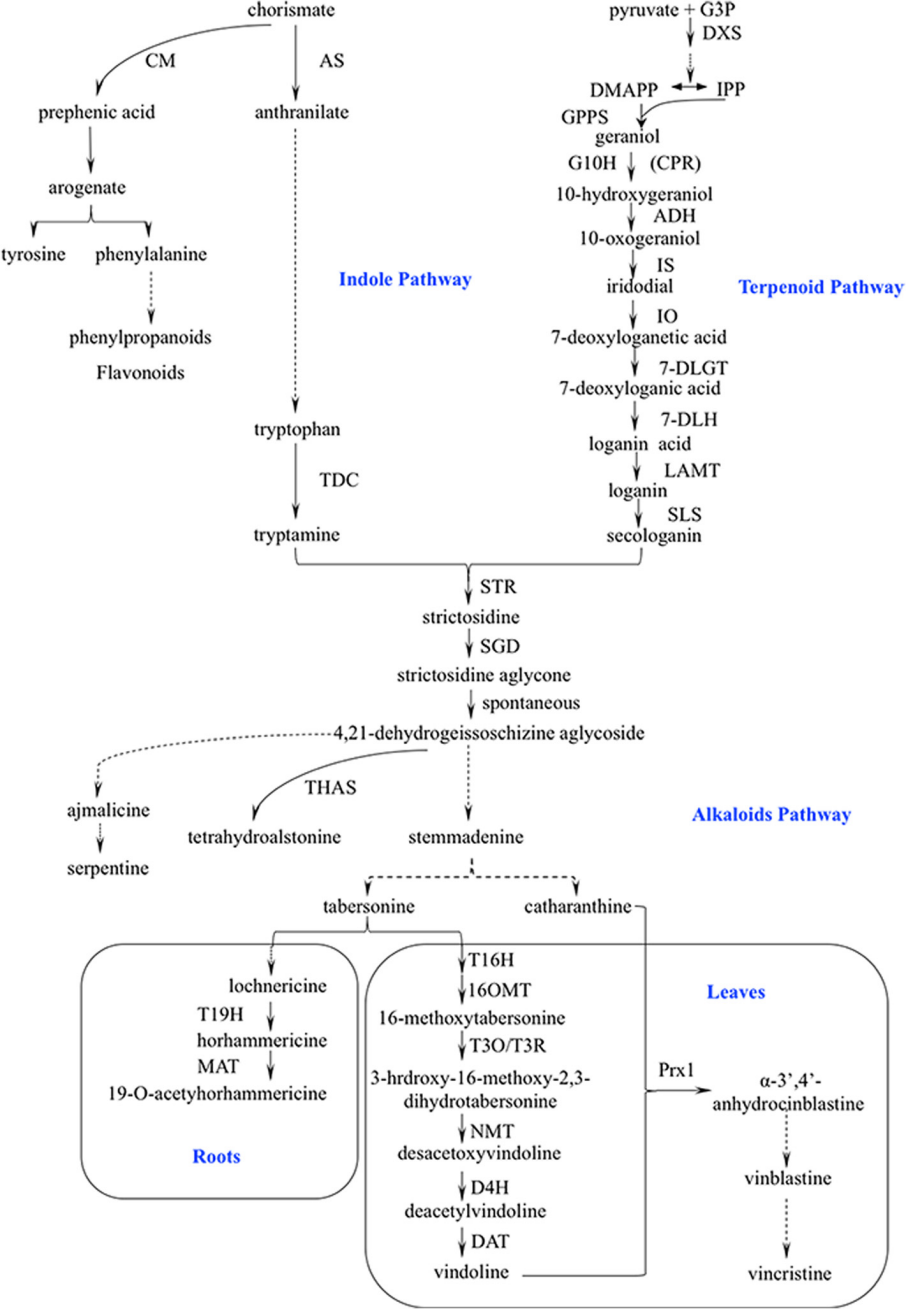
Terpenoid indole alkaloid pathway. (Dashed lines represent the unknown steps) [5, 47–50]

The medicinal plant *Catharanthus roseus* (Madagascar periwinkle) produces more than 130 identified terpenoid indole alkaloids (TIAs) [1]. Many of these TIAs are of great pharmaceutical importance. For example vinblastine and vincristine, which are exclusively synthesized in *C. roseus*, have been widely used clinically as anti-cancer agents to treat lymphoma and leukemia [2]. The TIA pathway leading to the biosynthesis of these pharmaceutically important compounds starts from the condensation of tryptamine and secologanin to form strictosidine [3]. Tryptamine is derived from shikimate and tryptophan biosynthesis pathway [4]. Secologanin is derived from MEP (2-C-methyl-D-erythritol 4-phosphate) and terpenoid pathway [5]. The first alkaloid strictosidine is converted to a wide range of TIAs through many branched downstream alkaloid pathways (Fig. 1). Some of the downstream pathways such as vindoline, hörhammericine, and catharanthine biosynthetic pathways are unique in *C. roseus* and have not been found in other organisms [6, 7]. However, *C. roseus* produces extremely low level of these pharmaceutical important TIAs [8]. Their complex structures constrain the economic feasibility of synthesis using chemical methods [9]. Thus, the pharmaceutical importance and the above challenges have motivated extensive studies to increase TIA production using genetic engineering in *C. roseus*.

Anthranilate synthase (AS) catalyzes chorismate to anthranilate, which is considered to be the rate-limiting step in indole pathway [10]. AS holoenzymes are heterotetramers composed of two alpha and two beta subunits. Of these two types of subunits, the alpha subunit is considered to play a crucial role in catalyzing chorismate to anthranilate. The binding site of tryptophan for feedback inhibition is present in the alpha subunit. The beta subunit possesses the amino-transferase activity, which transfers an amino group from glutamine to the alpha subunit [11]. Constitutive expression of ASβ subunit coupled with inducible overexpression of a feed-back resistant ASα subunit from *Arabidopsis* resulted in the increased concentration of tryptophan, tryptamine, and ajmalicine, while the concentration of lochnericine, hörhammericine, and tabersonine decreased over the 72 h induction period [12]. Feeding terpenoid precursor loganin to the AS overexpressing *C. roseus* hairy roots helped enhance the downstream alkaloids catharanthine (26 %), ajmalicine (84 %), lochnericine (119 %), and tabersonine (225 %) compared to unfed hairy roots overexpressing AS, but the increases are still limited compared to the increases in tryptophan (3000 %) [13]. Similarly, engineering other pathway genes [14, 15] or transcription factors [16, 17] achieved very limited success in increasing TIA accumulation. These results suggest that the TIA biosynthesis is under a tight regulation when the pathway gene was overexpressed.

TIA production is controlled at the transcriptional, translational and post-translational levels. The most studied regulation is transcriptional changes of the TIA biosynthetic genes by transcription factors in a coordinate manner in response to developmental and environmental signals such as jasmonate [18], fungal elicitors [19], salicylic acid [20], ethylene [21], nitric oxide (NO) [22], auxin [23], and cytokinins [24]. These molecules affect the TIA production synergistically or antagonistically through different signal transduction mechanisms [25]. Although extensive research has studied the effect of individual signaling molecules on TIA biosynthesis, the entire regulatory mechanism is not yet elucidated.

The metabolic burden caused by the significant accumulation of tryptophan and tryptamine when AS is overexpressed in *C. roseus* hairy roots could result in system wide transcriptional and metabolic changes similar to the responses seen in *Arabidopsis* and rice. High levels of expression of OASA1D (a feedback-insensitive alpha subunit of anthranilate synthase) in *Arabidopsis* resulted in increased concentrations of phenylalanine and tyrosine but decreased concentrations of their derived secondary metabolites, phenylpropanoids and flavonoids [26]. Enhanced AS activity in *Arabidopsis* induced the production of some indole derived secondary metabolites in response to exogenous stimuli [27, 28]. The OASA1D rice line had higher levels of anthranilate, tryptamine and serotonin compared to the wild type lines [29]. Transcriptomic analysis on OASA1D engineered rice by microarray resulted in the differential transcription of 2211 genes, most of which were categorized to the following cellular functions: cell wall, membrane and transport, cell processes and reproduction, energy flow, environmental response and metabolism and development [29]. Thus, we hypothesized that AS overexpression in *C. roseus* hairy roots would trigger global transcriptional change that can directly or indirectly affect TIA biosynthesis. In the present study, RT-qPCR is applied to examine changes in transcription of known TIA pathway genes and regulators due to the overexpression of AS. Furthermore, RNA-seq is utilized to further understand the global response of metabolic and regulatory networks when AS is overexpressed in *C. roseus* hairy roots. This RNA-seq study helps to increase the understanding of the regulation of TIA pathway and sheds light on rational metabolic engineering strategies to enhance TIA production in *C. roseus* hairy roots.

## Results

### ASα induced expression and TIA metabolites levels

**Fig. 2:**
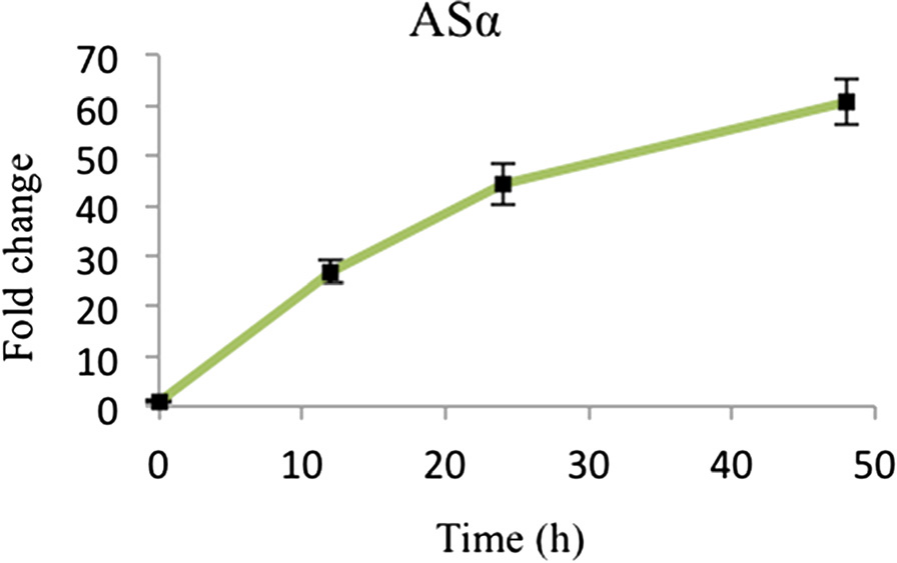
Fold change of transcript levels of ASα in the induced transgenic *C. roseus* hairy roots compared to the uninduced control at 12, 24 and 48 h. Data represents the mean of triplicate ± standard deviation.

In this study, we used a previously generated *C. roseus* hairy root line ASαβ-1 that carries an *Arabidopsis* feedback-resistant ASα subunit and a *C. roseus* ASβ subunit [10]. The expression of ASα is under the control of a glucocorticoid-inducible promoter, and ASβ is constitutively expressed under the CaMV 35S promoter. After 48 h, the transcripts of ASα demonstrate a 60 fold increase over the uninduced condition (Fig. 2). Additionally the overexpression of AS resulted in an increase in the concentrations of tryptophan, tryptamine and ajmalicine after 72 h induction, while tabersonine, lochnericine and hörhammericine concentrations decreased over the same period (Additional file 1: Figure S1) which is unfavorable. These results are consistent with a previous study [12]. The activity of anthranilate synthase in 72 h induced and uninduced hairy roots were measured and shown in Additional file 1: Figure S4 which indicates AS transcript correlates to AS activity levels.

### Transcriptional response of TIA genes and regulators by RT-qPCR

Although the genetic modification is targeted to one gene in the indole pathway, it may lead to unexpected transcriptional responses of other genes in the TIA pathway, which may constrain the metabolic flux toward downstream TIAs. The transcripts of a variety of TIA pathway genes and regulators were analyzed by RT-qPCR in the ASα induced and the un-induced ASαβ-1 hairy root line over a 48 h period.

**Fig. 3:**
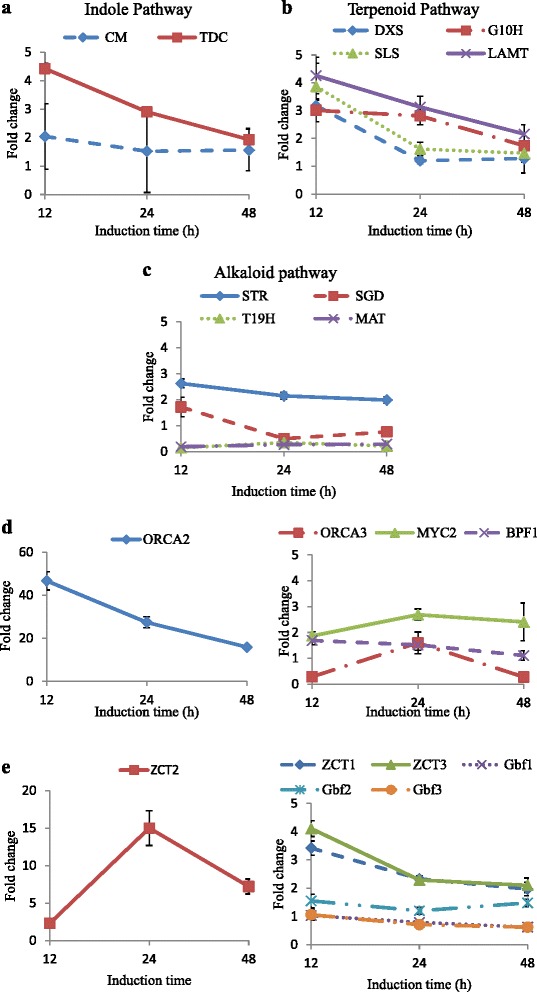
mRNA fold changes of indole (**a**), terpenoid (**b**), and alkaloid (**c**) pathway genes, positive regulators (**d**) and negative regulators (**e**) in the AS overexpressing hairy roots compared to the uninduced control at 12, 24, and 48 h of induction. Data represents the mean of triplicate ± standard deviation.

For the indole pathway (Fig. 3a), TDC (tryptophan decarboxylase) transcript levels showed the greatest up-regulation at 12 h. Then, this up-regulation was weakened and stabilized from 12 to 48 h. TDC encodes the last enzyme in the indole pathway converting tryptophan to tryptamine. CM (chorismate mutase) competitively uses the same precursor as AS and catalyzes chorismate to prephenate, which directs chorismate to an alternative pathway leading to the biosynthesis of phenylalanine. CM did not reveal significant change at the transcriptional level during the 48 h of AS overexpression. Within the terpenoid pathway (Fig. 3b), the transcript levels of the terpenoid genes DXS (1-deoxy-D-xylulose 5-phosphate synthase), G10H (geraniol 10-hydroxylase), SLS (secologanin synthase) and LAMT (loganic acid methyltransferase) reached the highest levels at 12 h followed by a decline to the uninduced levels by 48 h. DXS and SLS showed faster attenuation of the up-regulation than LAMT and G10H from 12 to 24 h induction.

For the alkaloid pathway (Fig. 3c), the first gene STR (strictosidine synthase), encoding the enzyme catalyzing the conversion of tryptophan and secologanin to the first alkaloid strictosidine, was up-regulated during the 48 h induction. The transcripts of SGD (strictosidine beta-glucosidase) were up-regulated at 12 h induction but were down-regulated at 24 h induction, and trended back to the uninduced level at 48 h induction. Interestingly, the downstream TIA genes T19H (tabersonine 19-hydroxylase) and MAT (minovincinine 19-hydroxy-O-acetyltransferase) showed significant down-regulation at 12, 24 and 48 h induction. This down-regulation could explain the decreases seen in lochnericine and hörhammericine concentrations after overexpressing AS.

The above results indicate the complex transcriptional response of the TIA pathway genes. Therefore, the mRNA levels of transcription factors of TIA pathway were measured for 48 h in the induced roots and the un-induced controls (Fig. 3d, e). The positive transcription factor ORCA2 (AP2-domain DNA-binding protein 2) was highly up-regulated from 12 to 48 h in the induced roots compared to the uninduced levels. ORCA3 (AP2-domain DNA-binding protein 3) showed down-regulation at 12 and 48 h after induction. The induced cultures showed a slight increase in BPF1 (box P-binding factor-1) and MYC2 transcripts levels compared to the uninduced cultures. The fold change of ORCA3, MYC2 and BPF1 are relative small compare to the fold change of ORCA2, which indicated ORCA2 played an important role after overexpressing AS. For the negative transcription factors, ZCT2 (zinc finger *Catharanthus* transcription factor) transcripts were highly up-regulated at 24 and 48 h. ZCT1 and ZCT3 showed an increase in up-regulation by 12 h induction. The GBF (G-box binding factor) transcription factors transcripts did not change over 48 h.

### Transcriptional response of overexpressing AS by RNA-seq

To further explore how the metabolic and regulatory pathways systematically change when overexpressing AS, the differential gene expression of uninduced and induced hairy roots line ASαβ-1 was conducted using next-generation, high-throughput sequencing of the transcriptome (RNA-seq). From RT-qPCR analysis, the highest transcriptional changes of the measured TIA pathway genes were mostly captured at 12 h and maintained at that level or trended back to control level, but some transcription factors reached their highest changes at 24 h induction of AS (Fig. 3), thus we choose to analyze the transcriptome of 18 h induced and uninduced hairy roots using RNA-seq expecting to capture majority of transcriptional changes in TIA related genes.

### *De novo* assembly and identifications of differentially expressed genes

**Fig. 4:**
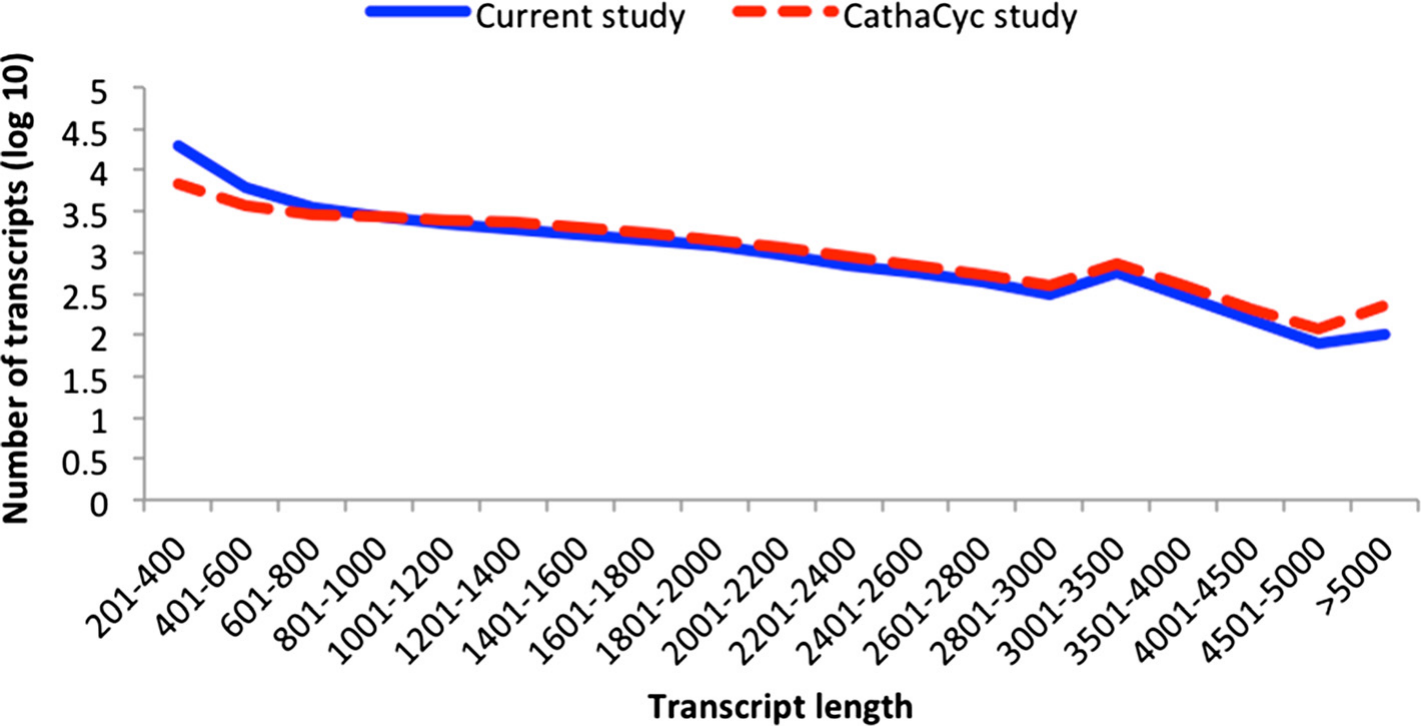
The length distribution of transcripts from trinity assembly and transcripts from public available *C. roseus* transcriptome data (http://www.cathacyc.org)

**Fig. 5:**
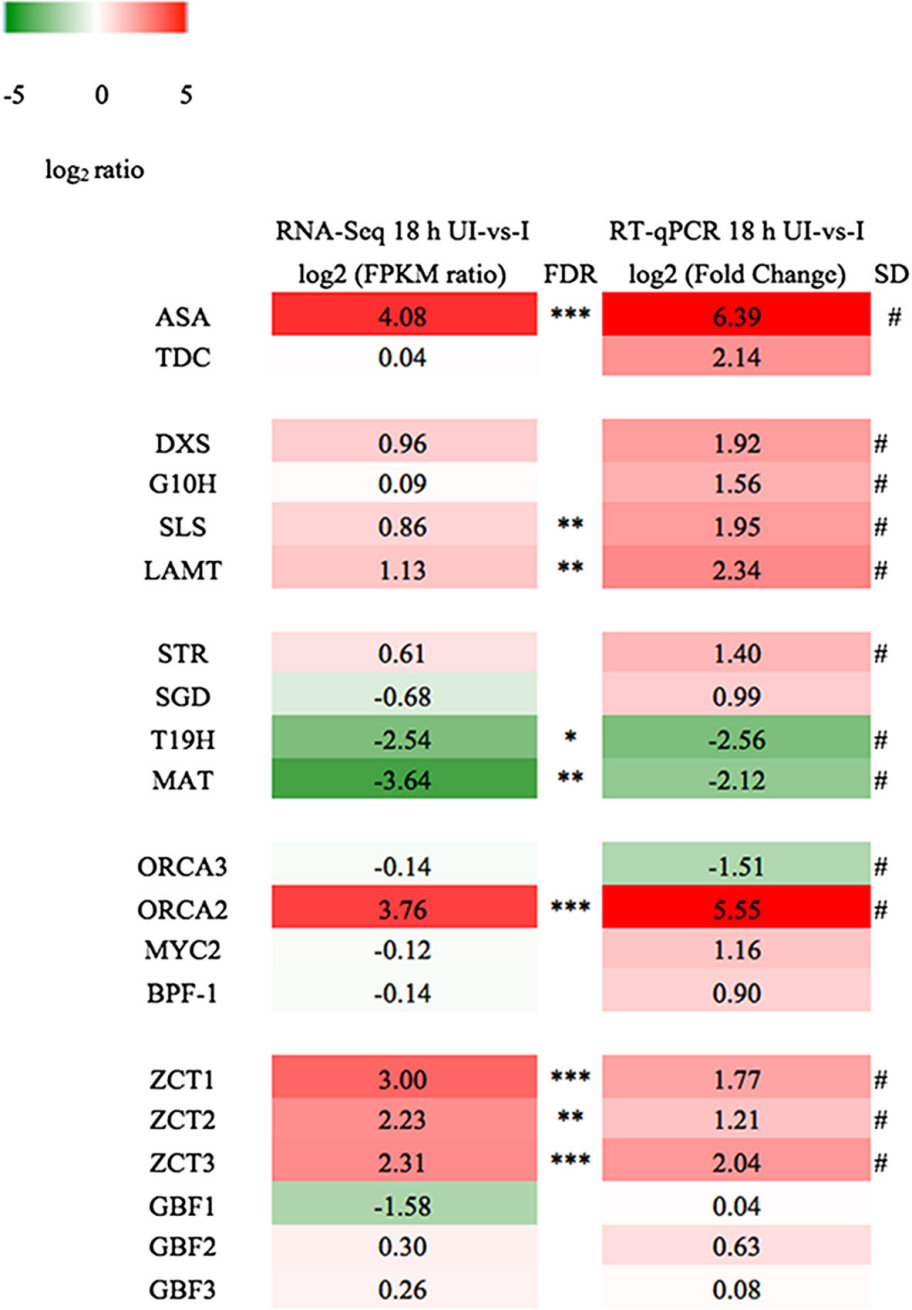
Log two ratios of relative expression levels or the FPKM (fragments per kilo base of exon per million fragments mapped) in the 18 h induced AS hairy roots compared to the control hairy roots by RT-qPCR and RNA-seq. “*” represents 10^-5^ < FDR < 10^-2^, “**” represents 10^-10^ < FDR < 10^-5^, “***” represents FDR < 10^-10^. “#” represents *p* < 0.05.

Total RNA with desired quality (RIN > 6.5, 28S:18S >1) and quantity (20 μg) was extracted from 18 h induced and uninduced hairy roots and was analyzed by high throughput sequencing. An average of 55 million clean reads (which were 99 % of raw reads) was obtained from each sample (SRA: SRP060820). The quality of the clean reads is shown in Additional file 1: Figure S2. The clean reads from all samples were assembled using Trinity methodology [30]. After Trinity assembling, 44,708 contigs (>200 bp) were obtained with a N50 length of 1418 nucleotides (nt) and an average length of 831 nt. This assembly resulted in a unigene set of 30,281, which was comparable to 31,450 unigenes in CathaCyc (a Metabolic Pathway Database Built from Catharanthus roseus RNA-Seq Data) [31]. The distribution of the lengths of assembled transcripts from Trinity method and from published CathaCyc is showed in Fig. 4 which indicates our assembly was similar to CathaCyc in respect to contig length and contig number. The translated protein sequences were used as queries to blast against *C. roseus* coding sequences database CathaCyc and resulted in 550 unique transcripts. Moreover, 39 genes out of the 43 known TIA pathway enzymes and regulators could be retrieved in the contig collections with minimum identity of 98 % of full length or near full length. Therefore, it supports the quality and potential utility of our sequencing and assembling data for downstream analysis. To avoid allelic differences causing complications for future downstream analyses, we used our assembled transcripts as the reference and followed the Trinity pipeline (https://github.com/trinityrnaseq/trinityrnaseq/wiki) to screen for differentially expressed genes (DEGs). In total, 2853 DEGs were generated in the 18 h AS overexpressing hairy roots compared to the uninduced control from RNA-Seq (Additional file 2: Table S2). There were 1341 up-regulated and 1512 down-regulated DEGs. Next, the changes in transcription of 20 TIA pathway genes and transcription factors in the 18 h induced and uninduced hairy roots were compared by RNA-Seq and RT-qPCR (Fig. 5). Most TIA pathway genes showed the same trend in both RNA-Seq and qPCR analysis which further validated the RNA-seq results. In addition, qPCR analysis of 18 h induced hairy roots captured all the transcriptional changed genes which were observed in Fig. 3. The transcriptional changes in ORCA2 and the ZCTs detected by RNA-Seq were also consistent with the qPCR results, indicating the active regulation of the TIA pathway by ORCA2 and the ZCTs.

### Gene Ontology and KEGG analysis

**Fig. 6:**
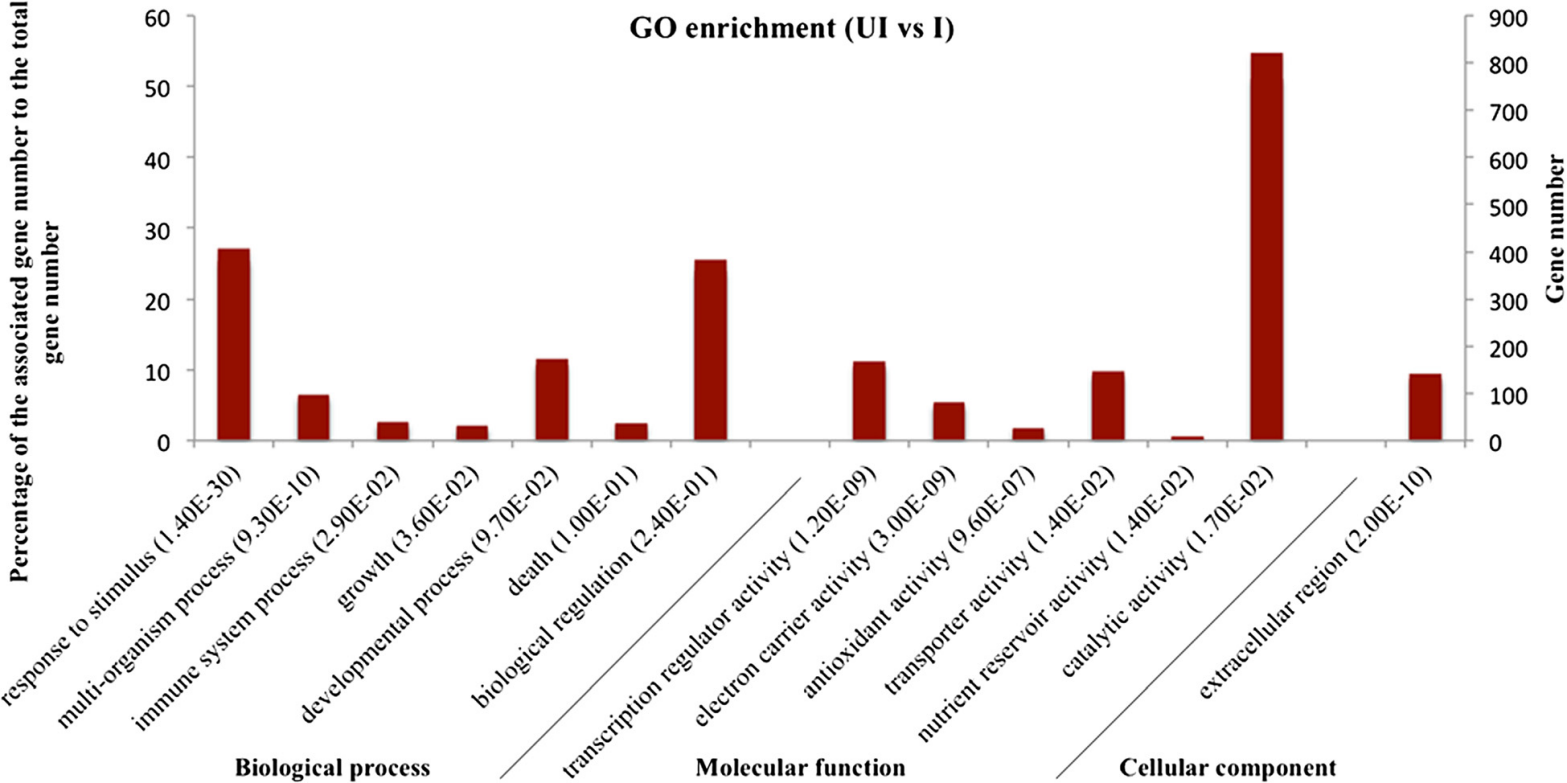
Enriched GO terms in DEGs compared to a total assembled transcripts reference as a background. The Benjamini adjusted *p* values were given in the bracket after each GO term.

**Fig. 7:**
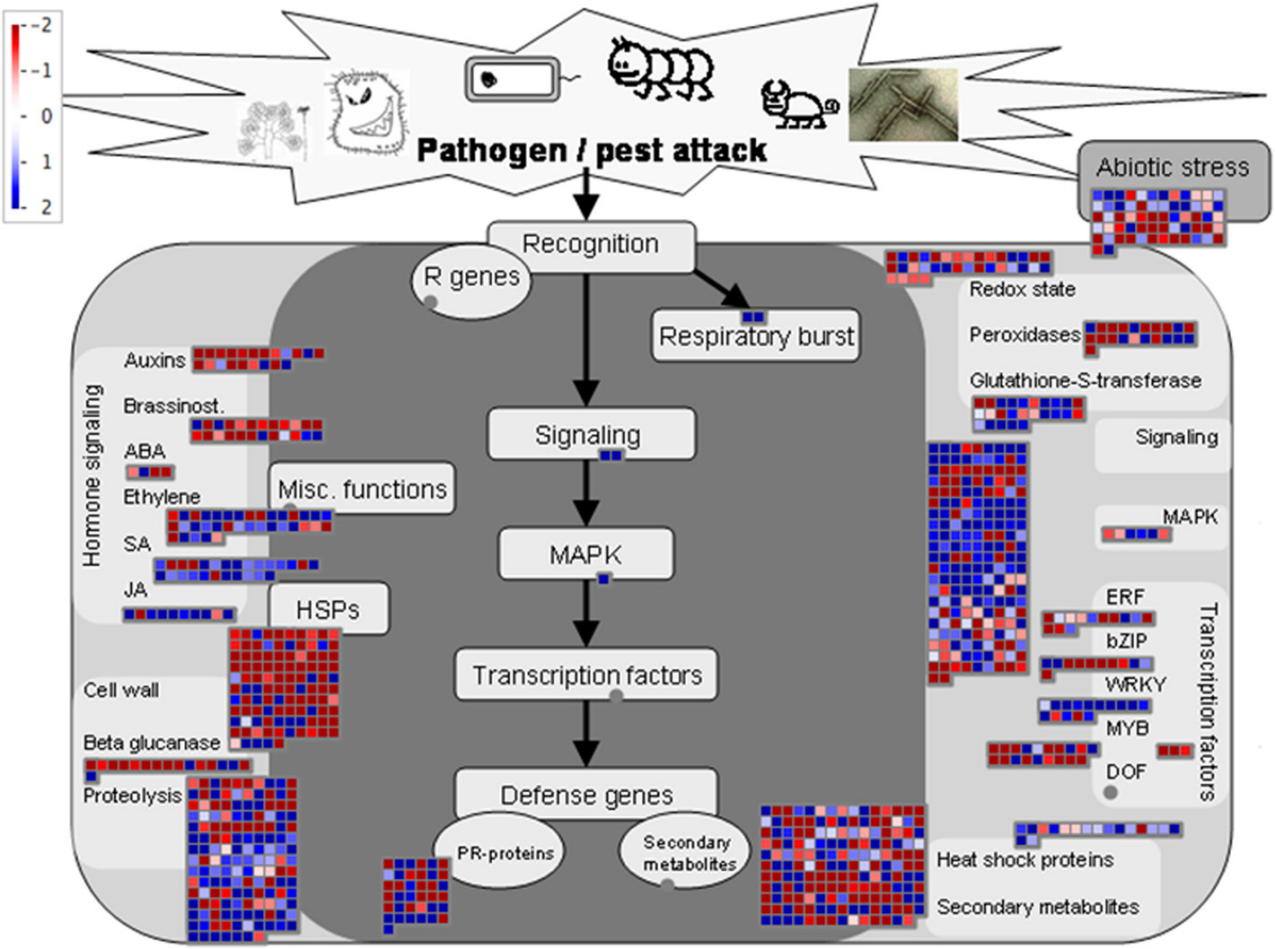
Stress response overview of transcriptome altered in response to overexpressing AS in *C. roseus* hairy roots by MapMan analysis. (http://mapman.gabipd.org)

A functional description for all assembled transcripts including DEGs was performed based on blastx analysis [32]. In total, 20,367 (67 %) unigenes with confidence e-value ≤ 10^−5^ were annotated against the UniProt database. Gene ontology (GO) assignments were used to classify the functions of the total assembled transcripts and the DEGs. GO terms that were significantly enriched in DEGs between the ASα induced verses un-induced conditions were shown corresponding to three categories in Fig. 6. In the category of biological process, response to stimulus was highly overrepresented with a *p*-value of 1.4x10^−30^ for DEGs compared to the transcriptome background. This suggests stress response was stimulated after overexpressing AS in *C. roseus* hairy roots. Moreover, multi-organism process was also enriched in DEGs with a *p*-value of 9.3x10^-10^. In the category of cellular component, DEGs mostly are present in extracellular region. In the category of molecular function, transcription regulator activity, electron carrier activity and antioxidant activity are all enriched in DEGs with *p*-values less than 10^−5^ (Fig. 6). The stress response was further visualized by MapMan analysis (Fig. 7). From Fig. 7, the DEGs involved in SA and JA signaling are mostly up-regulated (21 out of 26 and 8 out of 10, respectively) while the auxins and brassinosteroid signaling involved DEGs are mostly down-regulated (16 out of 21 and 18 out of 24, respectively). 304 signaling process involving genes and 468 TFs belongs to different groups were mapped with DEGs. This reveals a substantial stress related alteration of the transcriptome in response to AS overexpression.

To identify the biological pathways that are active in the AS overexpressing hairy roots, the DEGs were mapped to the reference canonical pathways in KEGG. The enriched pathways corresponding to the significant up and down regulated DEGs are listed in Tables 1 and 2. Biosynthesis of plant hormones, phenylpropanoid biosynthesis, and alkaloids biosynthesis were highly enriched pathways in both up and down regulated DEGs. Interestingly, phenylalanine, tyrosine and tryptophan biosynthesis, alpha-linolenic acid metabolism, fatty acid metabolism, glutathione metabolism, and tyrosine metabolism were significantly over-representative pathways in the up-regulated DEGs, while amino sugar and nucleotide sugar metabolism, starch and sucrose metabolism, glycolysis/gluconeogenesis, pyruvate metabolism and cysteine and methionine metabolism were identified in the down-regulated DEGs. This implies that diverse metabolic processes participate in the global response to the overexpression of AS in *C. roseus* hairy roots. Notably, alpha-linolenic acid metabolism ranks on the top enriched pathway for DEGs showing up-regulation (Table 1). Alpha-linolenic acid metabolism leads to the biosynthesis of an important hormone jasmonic acid which is involved in the up-regulation of the TIA pathway.

**Table 1:** The enriched pathways of significantly up-regulated DEGs

Pathway	Count	*p*-value	Adjusted *p*-value
Biosynthesis of plant hormones	19	7.90E-13	9.10E-11
Biosynthesis of phenylpropanoids	16	4.10E-12	2.40E-10
Biosynthesis of alkaloids derived from shikimate pathway	11	2.70E-08	1.00E-06
Phenylalanine, tyrosine and tryptophan biosynthesis	7	8.60E-08	2.50E-06
Stilbenoid, diarylheptanoid and gingerol biosynthesis	5	3.20E-05	7.20E-04
Phenylpropanoid biosynthesis	7	4.00E-05	7.70E-04
alpha-Linolenic acid metabolism	5	1.60E-04	2.70E-03
Endocytosis	6	2.50E-04	3.60E-03
Fatty acid metabolism	5	2.60E-04	3.40E-03
Biosynthesis of alkaloids derived from ornithine, lysine and nicotinic acid	7	2.70E-04	3.10E-03
Biosynthesis of alkaloids derived from histidine and purine	7	3.00E-04	3.10E-03
Methane metabolism	6	3.40E-04	3.20E-03
Glutathione metabolism	5	4.10E-04	3.60E-03
Biosynthesis of terpenoids and steroids	7	1.00E-03	8.40E-03
Tyrosine metabolism	4	1.20E-03	8.80E-03

**Table 2:** The enriched pathways of significantly down-regulated DEGs

Pathway	Count	*p*-value	Adjusted *p*-value
Biosynthesis of phenylpropanoids	18	8.60E-14	1.20E-11
Phenylpropanoid biosynthesis	13	2.20E-12	1.50E-10
Biosynthesis of plant hormones	19	4.90E-12	2.30E-10
Methane metabolism	12	2.80E-11	9.60E-10
Phenylalanine metabolism	11	2.40E-10	6.70E-09
Biosynthesis of terpenoids and steroids	13	3.60E-09	8.30E-08
Steroid biosynthesis	7	2.60E-08	5.10E-07
Biosynthesis of alkaloids derived from terpenoid and polyketide	8	4.90E-05	8.50E-04
Amino sugar and nucleotide sugar metabolism	7	5.10E-05	7.80E-04
Starch and sucrose metabolism	6	2.10E-04	2.90E-03
Glycolysis/Gluconeogenesis	6	3.00E-04	3.70E-03
Biosynthesis of alkaloids derived from ornithine, lysine and nicotinic acid	7	4.60E-04	5.30E-03
Pyruvate metabolism	5	6.00E-04	6.40E-03
Biosynthesis of alkaloids derived from shikimate pathway	7	6.40E-04	6.30E-03
Cysteine and methionine metabolism	5	1.00E-03	9.60E-03

## Discussion

Genetic and metabolic engineering techniques have enabled manipulation of the production of specific plant secondary metabolites of interest by modifying the genes that play a key role in the biosynthetic pathway. However, the metabolic pathway is a highly integrated network. Any perturbation in a given biosynthetic pathway is likely to cause a series of alterations in the transcription of the whole system. Those alterations may involve the plant’s regulatory system which is designed to tightly control secondary metabolite production. Frequently the mechanism for this regulation is poorly understood. Overexpressing the rate-limiting enzyme AS in the indole pathway within *C. roseus* hairy roots not only led to the transcriptional change of closely related TIA pathway genes, but also to the broader transcriptional changes ranging from primary to other secondary metabolite pathways. The 2853 differentially expressed transcripts were classified into different biological process and functions. Functional annotation of these DEGs helped elucidate processes involved in the response to overexpressing AS.

### TIA pathway changes after overexpressing AS

Both RT-qPCR and RNA-seq results showed AS modification perturbs transcription of many TIA pathway genes in *C. roseus* hairy roots. Overexpressing AS located in the upper indole pathway induced the transcription of the later indole pathway gene TDC and most measured terpenoid genes including DXS, G10H, SLS and LAMT. However, it had mixed effect on the transcription of alkaloid pathway genes. STR encoding the first committed enzyme in the alkaloid pathway was up-regulated while the downstream genes such as T19H and MAT were significantly down-regulated, which is different from the effect of jasmonic acid elicitation (data now shown here). Extensive studies showed that feeding jasmonic acid resulted in the up-regulation of all the known TIA pathway genes [33, 34]. It is hypothesized that the response to overexpressing AS might involve a different set of regulatory mechanisms than those involved in jasmonic acid transduction.

Overexpression of ASαβ gene altered the transcript levels of many transcription factors of TIA pathway (Figs. 3 and 5). The transcripts of positive regulator ORCA2 and negative transcription factor ZCT2 were greatly up-regulated indicating that these two transcription factors played an important role in this study. The expression of both the ORCA and ZCT TF families can be up-regulated by jasmonic acid and are believed to be involved in the jasmonate-inducible control of the TIA pathway genes. Feeding jasmonic acid led to the rapid up-regulation of TIA genes such as DXS, G10H, SLS, STR, SGD, AS, and TDC. The later attenuation of the up-regulation of TIA pathway genes with time was observed and was likely mediated through the combination effect of both positive and negative regulators, which can fine-tune the TIA biosynthesis to help the plant modulate their energy and resource balance between growth and defense [33]. Noticeable, ORCA3 was down-regulated at 12 and 48 h of AS overexpression which is opposite to ORCA3 up-regulation in response to JA feeding. The regulation of TIA genes and the targets of each transcription factors are still far from being understood.

Although genetic engineering of AS led to a large increase in tryptophan and tryptamine, the changes in TIA concentrations are relatively small. The enhanced transcriptions of both positive and negative regulators of the pathway were observed which can counterbalance to help the plant maintain homeostasis of alkaloids concentrations. The highest transcriptional change of the measured TIA pathway genes and transcription factors usually occurred at 12 h or 24 h after induction of AS. These transcriptional changes were diminished with time and trended back to the uninduced level. All together, these results indicate the complex, dynamic and tight regulation of TIA biosynthesis in *C. roseus* hairy roots. A poor understanding of this regulation means that it is challenging to use genetic engineering to enhance these clinically useful TIAs. In other AS engineered plants, this kind of tight regulation is also observed. In OASA1D overexpressed rice calli, no over-accumulation of secondary metabolites derived from the tryptophan pathway was observed except for a novel indole compound derived from indole glycerol-3-phosphate [35]. Metabolic profiling of OASA1D modified rice revealed no substantial changes in the amounts of other phenolic compounds except for two fold increase in indole acetic acid in the seeds of the transgenic lines [36]. Analysis of tryptophan distribution in OASA1D rice and *Arabidopsis* revealed accumulation of tryptophan occurred at highest concentration in newly formed tissues which suggest that the plant had the capacity to translocate excess tryptophan from source organs to reproductive organs. These results clearly pointed that the secondary metabolites were strictly regulated at transcriptional and transportation levels and proceeded in an orderly manner even when a greater supply of tryptophan was available by overexpressing feedback insensitive AS.

### Aromatic amino acid biosynthetic pathway alterations after overexpressing AS

Manipulation of the AS gene in tryptophan biosynthesis pathway in *C. roseus* also causes changes of multiple pathways interacting directly or indirectly with the tryptophan biosynthesis pathway. An important directly related pathway is the pathway competing for common precursors. All three aromatic amino acids are synthesized via the shikimate pathway followed by the branched aromatic amino acid metabolic pathway, with chorismate serving as a common precursor. AS converts chorismate to anthranilate leading to the tryptophan production, while CM catalyzes chorismate to prephenate that serves as a precursor for the biosynthesis of phenylalanine and tyrosine. The enhanced activity of a target pathway usually results in the decrease in substrate supply to a competing pathway. In AS overexpressing *C. roseus* hairy roots, tryptophan biosynthesis pathway was activated and tryptophan accumulation was increased (Additional file 1: Figure S1). From pathway enrichment analysis, phenylalanine, tyrosine and tryptophan biosynthesis were highly enriched in up-regulated DEGs (Table 1), which indicated the concentration of the other two aromatic amino acid phenylalanine, and tyrosine could also be increased. Many up-regulated transcripts were mapped to shikimate pathway (Additional file 1: Figure S3) which may lead to an increase in the common precursor chorismate supply. Furthermore, the regulation of the aromatic amino acid biosynthesis is complex and far from being understood. In the model plant system *Arabidopsis*, chorismate mutase of phenylalanine and tyrosine synthesis proved experimentally to be allosterically regulated. In *C. roseus*, only chorismate mutase like CrUnigene has been reported [37]. The transcriptional level of this transcript remained unchanged after overexpressing AS, but it is very likely regulated by the change of conformation in *C. roseus*. In *Arabidopsis*, rice and other plants, tryptophan activates CM activity while phenylalanine and tyrosine inhibit CM activity. The overproduction of tryptophan in AS engineered hairy roots could possibly increase the metabolic flux to the phenylalanine and tyrosine biosynthesis through the activation of CM.

Overexpressing AS directly increased the tryptophan level that provided the precursor for TIA biosynthesis. The up-regulation of the other two aromatic amino acids (phenylalanine and tyrosine) biosynthesis can provide precursors for a wide range of secondary metabolites. Phenylalanine serves as the precursor for the phenylpropanoid which is an essential component of a number of structural polymers, provide protection from ultraviolet light, defend against herbivores and pathogens, and mediate plant-pollinator interactions as floral pigments and scent compounds [38]. Pathway enrichment analysis showed phenylpropanoid biosynthesis pathway was significantly altered (Tables 1 and 2) which indicates overexpressing AS can also change a variety of secondary metabolites biosynthetic genes transcription not limited to TIAs.

### Stress responses after overexpressing AS

Response to stimulus was the most significantly enriched biological process in DEGs by GO enrichment study (Fig. 6). From Fig. 7, a substantial transcriptional alteration in regard to plant stress response was observed in response to overexpressing AS. TIAs, acting as toxins to the attacking organism, involved in direct defense against abiotic and non-abiotic plant stress in *C. roseus*. Many developmental and environmental signals such as fungal elicitors, jasmonate, salicylic acid, ethylene, and nitric oxide can trigger a series of plant hormone transduction pathways followed by mitogen-activated protein kinase (MAPK) cascade and change the expression of multiple transcription factors leading to the increased accumulation of TIAs [39]. Among these phytohormones, salicylic acid and jasmonic acid are extensively studied and proved to synergistically affect TIA production, while plant growth hormones such as auxins negatively influence transcriptional level of the TIA pathway genes [4]. Based on overexpressing AS in *C. roseus* hairy roots, the transcripts involving in SA and JA signaling are mostly up-regulated while the auxins and brassinosteroid signaling related transcripts were mostly down-regulated (Fig. 7). However, the regulatory mechanism and the crosslink between these hormone transduction pathways are still poorly understood in *C. roseus*. Further pathway enrichment analysis showed biosynthesis of plant hormones is the top ranked enriched pathway based on AS overexpression (Tables 1 and 2). Notably, alpha-linolenic acid metabolism, which can lead to jasmonic acid biosynthesis, was the significantly enriched pathway of up-regulated DEGs (Table 1). Other hormones biosynthesis pathways were not significantly altered by pathway enrichment analysis. This demonstrates jasmonic acid played an important role in regulating TIAs and other secondary metabolites biosynthesis after overexpressing AS. Moreover, some primary pathways related to plant growth including amino sugar and nucleotide sugar metabolism, starch and sucrose metabolism, glycolysis/gluconeogenesis, pyruvate metabolism and cysteine and methionine metabolism were enriched corresponding to down-regulated DEGs. Thus, overexpressing the indole pathway gene AS triggered the overall stress response in plant, and this stress response was likely mediated in part through the increased biosynthesis of jasmonic acid. Under this stress conditions, *C. roseus* regulated its primary metabolites and secondary metabolites biosynthesis to balance between growth and defense.

## Conclusion

**Fig. 8:**
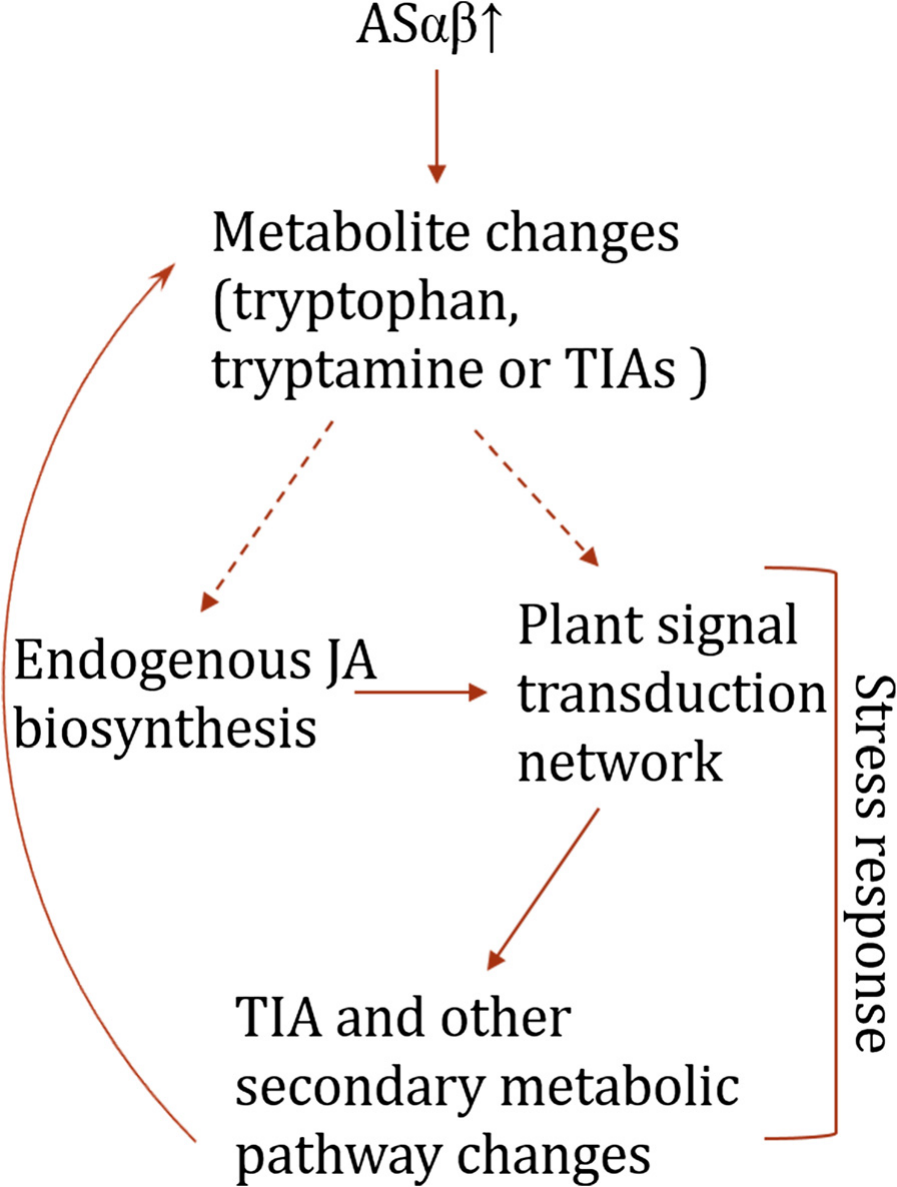
The overview of effect on overexpressing AS in *C. roseus* hairy roots. Overexpressing AS caused the changes of direct metabolite levels including tryptophan and tryptamine. The endogenous JA biosynthesis pathway genes were upregulated and multiple plant hormone transduction pathway were triggered, which can regulate TIA and other secondary pathway genes transcription. The transcriptional changes in secondary pathway genes will in turn change the TIA and other secondary metabolites accumulations.

This study provided a global analysis of transcriptome from AS transgenic *C. roseus* hairy root and serve as an available resource of genetic diversity. TIA biosynthesis is a tightly coordinated process. Changes in TIA metabolite concentrations in response to external stimuli or genetic modification tend to be limited in magnitude, while the changes in TIA transcripts happen within the first 24 hours before returning back to pre-stimuli levels. This study reports a comprehensive transcriptomic analysis of *C. roseus* hairy roots overexpressing the rate-limiting enzyme AS in the indole pathway. The use of various bioinformatics tools identified highly involved pathways and cellular processes when AS was overexpressed. RT-qPCR validated the altered transcription of TIA pathway related genes. The overall stress responses were stimulated in the AS engineered hairy roots which is reflected by the differential expression of genes related to plant-pathogen interactions (Fig. 8). Particularly, up-regulation of endogenous JA biosynthesis pathway signified the involvement of JA signal transduction to regulate TIA biosynthesis in AS engineered roots (Fig. 8). However, whether the stress response was resulted from the increased accumulation of tryptophan or other metabolite changes remains unclear (Fig. 8). Furthermore, the down-regulation of T19H and MAT after overexpressing AS suggests jasmonic acid is not the solely regulating mechanism participating in response to overexpressing AS in *C. roseus* hairy roots. The global transcriptional alterations of this investigation open new insight into the complex regulation of TIA pathway in AS overexpressing *C. roseus* hairy roots.

## Methods

### Hairy roots material

The media for hairy roots growing is a filter-sterilized solution of 30 g/L sucrose, half-strength Gamborg’s B5 salts (Sigma-Aldrich) and full-strength Gamborg’s vitamins (Sigma-Aldrich) adjusted to a pH of 5.7. Hairy roots cultures were initiated by placing five root tips into a 250 mL flask with 50 mL of media and grown in the dark at 26 °C and 100 rpm. Roots were subcultured every three weeks. ASαβ-1 hairy root line was a gift from Ka-Yiu San’s lab at Rice University. The generation of ASαβ-1 hairy root line was described by Hong *et al.* [40]. This hairy root line constitutively expresses ASβ subunit from *Arabidopsis* under CaMV 35S promoter and transiently expresses the feedback-resistant ASα subunit from *Arabidopsis* under the control of a glucocorticoid-inducible promoter.

### Induction study and alkaloids measurement

To study the inducible expression of ASαβ, ASαβ hairy roots were treated with 0.2 μM dexamethasone (induced cultures) or with an equal amount of ethanol (uninduced cultures) as a negative control on day 18 of the growth cycle. Three flasks of induced and three flasks of uninduced hairy roots were harvested at 12, 24 and 48 h after dexamethasone or ethanol treatment. In addition, three flasks were harvested with treatment of ethanol for 0 h as a control. Three hundred milligram of fresh weight tissue was ground in liquid nitrogen and stored at -80 °C for further mRNA analysis. The remaining tissue was harvested for alkaloid metabolite detection. Alkaloids were extracted from the freeze-dried hairy roots using methanol. The concentration of alkaloids was detected by HPLC (SHIMADZU, Japan) following previously described protocols [12].

### cDNA synthesis and RT-qPCR amplification

The total RNA extraction and reverse transcription to cDNA was previously described [33]. A no amplification control lacking reverse transcriptase was performed for each sample. The first strand cDNA was diluted 10 times to a final volume of 200 μL with nuclease-free water. PCR amplification was carried out in a 96 well plate on CFX96 Touch^TM^ Real-Time PCR detection system (BIORAD, USA). Each reaction contained a mixture of 1 μL diluted cDNA (which corresponds to 500 mg of total RNA), 9 μL mixed primers (1.25pmol/mL), and 10 μL SYBR Green PCR Master Mix (BIORAD). The primers used which were not previously described [33] were listed in Additional file 1: Table S1. The reaction mixture was incubated for 30 s at 95 °C, and for 40 cycles of 15 s at 95 °C and 30 s at 60 °C [33]. The relative gene transcription was quantified by using the comparative C_T_ (threshold cycle) method as previously described [41]. Briefly, the relative transcription of measured gene in the induced hairy roots were shown by 2^(-ΔΔC_T_) normalizing to the control gene (40S ribosomal protein S9) and the transcription level in the uninduced control [42].

### RNA preparation and Illumina sequencing

The *C. roseus* hairy roots treated with the inducer and the ethanol for 18 h were chosen to do the RNA-seq study. The total RNA samples were extracted using Trizol (Invitrogen). 6 ml of Trizol was used to treat 100 mg of flash frozen hairy roots. RNA extracts were treated twice with DNaseI (Invitrogen) to ensure removal of all DNA. For quality control, Agilent 2100 Bioanaylzer and ABI StepOnePlus Real-Time PCR System were used to qualify and quantify the sample library. Twenty micrograms of RNA was sent to BGI Americas (http://bgiamericas.com/) for library construction and for high throughput sequencing. The mRNA is enriched by using the oligo (dT) magnetic beads. Mixed with the fragmentation buffer, the mRNA is fragmented into short fragments (about 200 bp). Then the first strand of cDNA is synthesized by using random hexamer-primers. Buffer, dNTPs, RNase H and DNA polymerase I are added to synthesize the second strand. The double strand cDNA is purified with magnetic beads. End reparation and 3’-end single nucleotide A (adenine) addition is then performed. Finally, sequencing adaptors are ligated to the fragments. The fragments are enriched by PCR amplification. The library was sequenced via Illumina HiSeqTM 2000.

### Raw data preprocess

Clean reads (SRA: SRP060820) were obtained by removing adaptor sequences, reads in which the percentage of unknown bases (N) is greater than 10 % and low quality reads (reads with the percentage of the low quality base (base with quality value ≤ 5) greater than 50 %).

### De novo assembly and abundance analysis

After successful high-throughput sequencing and quality filtering, the RNA-seq clean reads from all samples were assembled using the short-read assembling program Trinity (https://github.com/trinityrnaseq/trinityrnaseq/wiki) [30]. In brief, contigs were obtained based on the overlapping of short sequences. Trinity integrates three software packages (Inchworm, Chrysalis, and Butterfly) to partition the sequences into individual de Bruijn graphs, each representing the transcriptional complexity at a given gene or locus, processes each graph to extract full-length splicing isoforms, and to tease apart transcripts derived from paralogous genes. Clean reads from each sample were mapped to the Trinity-assembled contigs using Bowtie 1 [43], then RSEM [44] was applied to estimate the abundance FPKM (fragments per kb of exon per million fragments mapped). The differential expression genes (DEGs) were screened out using edgeR [45] with a cutoff of FDR (false discovery rate) ≤0.05 and minimum sum account 5.

### Functional annotation

Assembled contigs from the two root conditions longer than 300 bps were treated with CD-HIT-est to reduce redundancy [46]. The transcripts were functionally annotated using blastx against Uniprot (http://www.uniprot.org/), GO (gene ontology) (http://geneontology.org/) and KEGG (Kyoto Encyclopedia of Genes and Genomes) (http://www.genome.jp/kegg/) databases based on a guilt by-homology approach (E-value ≤ 10^−5^). GO and KEGG enrichment analysis provides all GO terms or pathways that significantly enriched in DEGs comparing to the transcriptome background. This method firstly maps all DEGs to GO terms or pathways in the database, calculating gene numbers for every term or pathway, then using hypergeometric test followed by Benjamini correction with a significance level of P < 0.01 to find significantly enriched GO terms and pathways in DEGs comparing to the transcriptome background. The enrichment analysis was performed using DAVID Bioinformatic resources 6.7 (https://david.ncifcrf.gov/summary.jsp). Mapman (http://mapman.gabipd.org/web/guest/mapman) was applied to visualize the pathway analysis results of DEGs.

### Availability of Supporting Data

The Illumina HiSeqTM 2000 sequencing data of the induced and uninduced ASαβ-1 *C. roseus* hairy roots in this study is available in SRA (Sequence Read Archive) repository [SRA: SRP060820, http://www.ncbi.nlm.nih.gov/sra/SRX1093623[accn] .

## Additional files

Additional file 1: Table S1. Primers for qRT-PCR. Figure S1. TIA metabolites. Figure S2. Per base quality scores for sequencing reads after quality filtering by the FastQC analysis. Figure S3. The aromatic amino acid pathway mapped with up-regulated DEGs. Figure S4. Enzyme activity of anthranilate synthase. (DOCX 150 kb) Additional file 2: Table S2. List of differentially expressed genes. (XLS 648 kb)

## Abbreviations

16OMT: 16-O-methyltransferase
7DLGT: 7-deoxyloganetic acid-O-glucosyl transferase
7DLH: 7-deoxyloganic acid hydroxylase
ADH: alcohol dehydrogenase
ASαβ: anthranilate synthase alpha beta subunits
BPF-1: box P-binding factor-1
CM: chorismate mutase
CPR: cpr mRNA for NADPH--ferrihemoprotein reductase
D4H: desacetoxyvindoline 4-hydroxylase
DAT: deacetylvindoline-4-O-acetyltransferase
DEGs: differentially expressed genes
DMAPP: dimethylallyl pyrophosphate
DXS: 1-deoxy-D-xylulose 5-phosphate synthase
FPKM: fragments per kb of exon per million fragments mapped
G10H: geraniol 10-hydroxylase
GBF: G-box binding factor
GO: gene ontology
IO: iridoid oxidase
IPP: isopentenyl pyrophosphate
IS: iridoid synthase
JA: jasmonic acid
KEGG: Kyoto Encyclopedia of Genes and Genomes
LAMT: loganic acid methyltransferase
MAT: minovincinine 19-hydroxy-O-acetyltransferase
MYC2: MYC2 type basic Helix-Loop-Helix (bHLH) transcription factor
NMT: N-methyltransferase
ORCA: AP2-domain DNA-binding protein
Prx1: Peroxidase 1
SGD: strictosidine beta-glucosidase
SLS: secologanin synthase
STR: strictosidine synthase
T16H: tabersonine 16-hydroxylase
T19H: tabersonine 19-hydroxylase
T3O: tabersonine 3-oxygenase
T3R: tabersonine 3-reductase
TDC: tryptophan decarboxylase
THAS: tetrahydroalstonine synthase
TIA: terpenoid indole alkaloid
ZCT: zinc finger *Catharanthus* transcription factor
