# Supplementary material for: Examining the transcriptional response of overexpressing anthranilate synthase in the hairy roots of an important medicinal plant Catharanthus roseus by RNA-seq
Source: BMC Plant Biol. 2016 May 6;16:108. doi: 10.1186/s12870-016-0794-4 (PMC4859987; doi:10.1186/s12870-016-0794-4)
Supplement: Additional file 1: Table S1. — Primers for qRT-PCR. Figure S1. TIA metabolites. Figure S2. Per base quality scores for sequencing reads after quality filtering by the FastQC analysis. Figure S3. The aromatic amino acid pathway mapped with up-regulated DEGs. Figure S4. Enzyme activity of anthranilate synthase. (DOCX 150 kb) [file 12870_2016_794_MOESM1_ESM.docx]

Table S1. Primers for qRT-PCR

| Gene | Enzyme of function | Primer pairs | PCR product (bp) |
| --- | --- | --- | --- |
| *AS*α | Anthranilate synthase  (M92353) | 5’-AGATCGTGGCAAAGGAGAAT-3’  5’-GCATCTGGAAGGTCCTGAAC-3’ | 150 |
| *LAMT* | loganic acid methyltransferase (EU057974) | 5’-CCAATGAAAGGTGGTGATGA-3’  5’-AATGCGGAAAGGTTTGATTG-3’ | 159 |
| *T19H* | tabersonine/lochnericine 19-hydroxylase (HQ901597.1) | 5’- CTTCATTTGCAATCCCCATT-3’  5’- AAACGAGAGAGGGTTTTGG-3’ | 150 |
| *Gbf3* | G-box binding factor bZIP transcription factor ([AY027510](http://www.ncbi.nlm.nih.gov/entrez/viewer.fcgi?db=nucleotide&val=13236839)) | 5’-GCTTCCACTGTTGCTTCTCC-3’  5’-CCTGGAGTCGTTGCCATAGT-3’ | 149 |
| *BPF1* | MYB-like DNA-binding protein ([AJ251686](http://www.ncbi.nlm.nih.gov/entrez/viewer.fcgi?db=nucleotide&val=12043532)) | 5’-CCAATGATGCATTTGATTCG-3’  5’-TGCAGGAAGAGTGACCAGTG-3’ | 143 |
| *LAMT* | loganic acid methyltransferase (EU057974) | 5’-CCAATGAAAGGTGGTGATGA-3’  5’-AATGCGGAAAGGTTTGATTG-3’ | 159 |
| *MAT* | minovincinine 19-hydroxy-O-acetyltransferase (AF253415.1) | 5’-AGGATTGGGCTGCTTCTACA-3’  5'- TATGGCTTCCGGAGAGAAGA -3' | 167 |
| *MYC2* | an early jasmonate-responsive bHLH transcription factor (AF283507) | 5’-CTGGGTTCAACGGAATTGAT-3’  5’-CGATGGATCAGTAAGCCACA-3’ | 147 |
| *CM* | Chorismate mutase (FD419102.1) | 5’-GGGTGATGCCTTTAACCAAA-3’  5’-GCGACATTGTATCGTGAAGC-3’ | 159 |
| *WRKY1* | Catharanthus roseus WRKY1 mRNA (HQ646368.1) | 5’-GAAACTCTCGCCGTACTTGG-3’  5’-CCGAAACATTCCTTCGTTTG-3’ | 159 |

Figure S1. TIA metabolites. Induced over-expression of ASαβ gene (▲, solid line) caused the greatest increase in tryptophan and tryptamine and slightly increase in ajmalicine, in contrast, caused a decrease in tabersonine, lochnericine and hörhammericine compare to the uninduced (×, dashed line) levels.


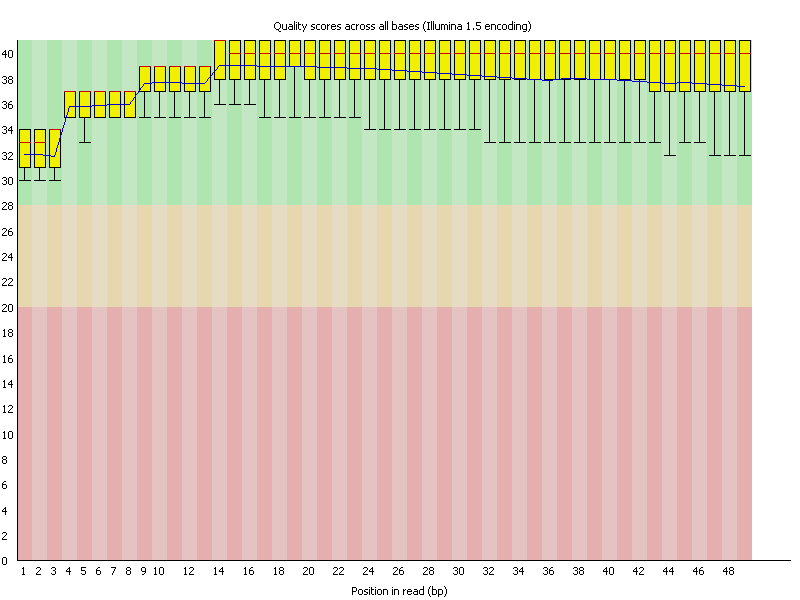


(a)


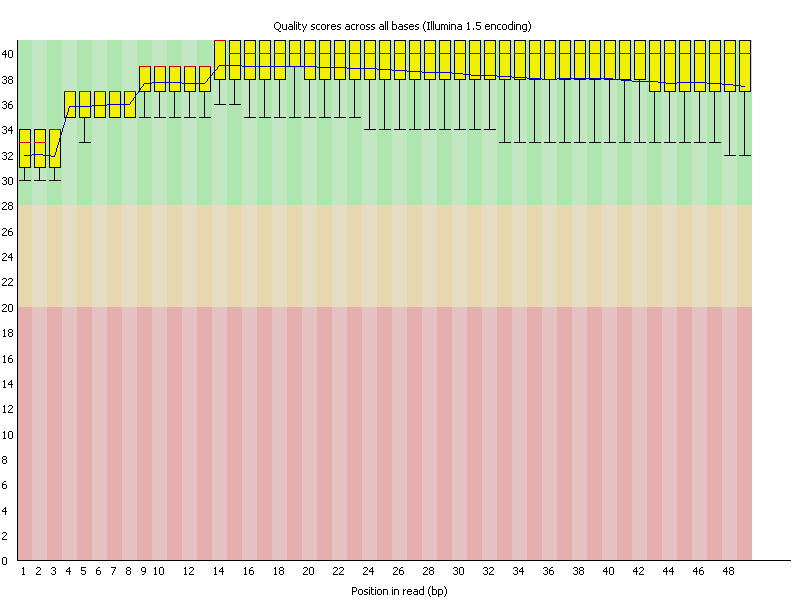


(b)

Figure S2. Per base quality scores for sequencing reads after quality filtering by the FastQC analysis of 18 h uninduced (upper) and induced (lower) C. roseus hairy roots. Central red line is the median value. Yellow box represents the inter-quartile range (25-75%). Upper and lower whiskers represent the 10% and 90% points. Blue line represents the mean quality.


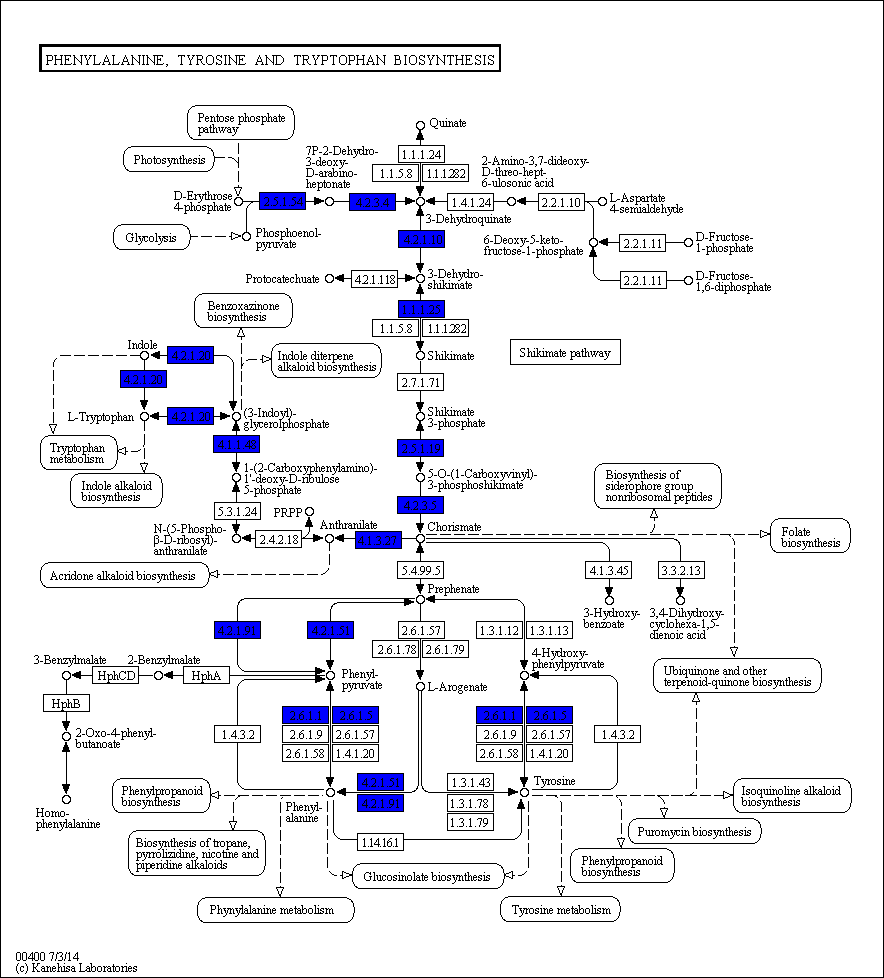


Figure S3. The aromatic amino acid pathway mapped with up-regulated DEGs. Blue boxes represent the gene mapped with the up-regulated DEGs. (<http://www.genome.jp/kegg/tool/map_pathway2.html>)

Figure S4. Enzyme activity of anthranilate synthase (AS) in AS transgenic *C. roseus* hairy roots was measured as previously described (Peebles et al., 2009). Triplicate cultures were induced with 0.2 mM dexamethasone (Induced) or an equal volume of ethanol (Uninduced) and harvested 72 h later.

**References**

Peebles, C.A., Sander, G.W., Li, M., Shanks, J.V., San, K.Y. 2009. Five year maintenance of the inducible expression of anthranilate synthase in Catharanthus roseus hairy roots. Biotechnol Bioeng 102:1521-1525.
